# Regulating the Porosity and Bipolarity of Polyimide‐Based Covalent Organic Framework for Advanced Aqueous Dual‐Ion Symmetric Batteries

**DOI:** 10.1002/advs.202407073

**Published:** 2024-08-19

**Authors:** Dongxiang Geng, Heng Zhang, Zhijian Fu, Ziming Liu, Yafei An, Jun Yang, Dawei Sha, Long Pan, Chao Yan, ZhengMing Sun

**Affiliations:** ^1^ School of Materials Science and Engineering Jiangsu University of Science and Technology Zhenjiang 212100 P. R. China; ^2^ Key Laboratory of Advanced Energy Materials Chemistry (Ministry of Education) Nankai University Tianjin 300071 P. R. China; ^3^ Institute of Technology for Carbon Neutralization Yangzhou University Yangzhou 225009 P. R. China; ^4^ School of Materials Science and Engineering Southeast University Nanjing 210089 P. R. China

**Keywords:** aqueous dual‐ion battery, bipolarity, covalent organic frameworks, hierarchical pore structure, high energy density

## Abstract

The all‐organic aqueous dual‐ion batteries (ADIBs) have attracted increasing attention due to the low cost and high safety. However, the solubility and unstable activity of organic electrodes restrict the synergistic storage of anions and cations in the symmetric ADIBs. Herein, a novel polyimide‐based covalent organic framework (labeled as NTPI‐COF) is constructed, featured with the boosted structure stability and electronic conductivity. Through regulating the porosity and bipolarity integrally, the NTPI‐COF possesses hierarchical porous structure (mesopore and micropore) and abundant bipolar active centers (C═O and C─N), which exhibits rapid dual‐ion transport and storage effects. As a result, the NTPI‐COF as the electrodes for ADIBs deliver a high reversible capacity of 109.7 mA h g^−1^ for Na^+^ storage and that of 74.8 mA h g^−1^ for Cl^–^ storage at 1 A g^−1^, respectively, and with a capacity retention of 93.2% over 10 000 cycles at 10 A g^−1^. Additionally, the all‐organic ADIBs with symmetric NTPI‐COF electrodes achieve an impressive energy density of up to 148 W h kg^−1^ and a high power density of 2600 W kg^−1^. Coupling the bipolarity and porosity of the all‐organic electrodes applied in ADIBs will further advance the development of low‐cost and large‐scale energy storage.

## Graphical abstract

A bipolar polyimide‐based covalent organic framework featured with hierarchical pore structure and high electronic conductivity is well‐designed and constructed, achieving a high‐performance dual‐ion cooperative storage capability in symmetric aqueous dual‐ion batteries.

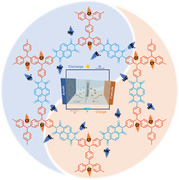

## 1 Introduction

The large‐scale energy storage puts more extreme demands on devices, such as high safety, low cost, long lifespan, and high energy density. Among various energy storage systems, the rechargeable lithium batteries possess high output voltage and energy density, playing a predominant role in the field of modern energy storage.^[^ ^1^, ^2^ ^]^ Whereas the high reactivity of lithium metal itself, as well as the flammability and toxicity of organic electrolytes, have triggered a series of safety issues.^[^ ^3^ ^]^ Therefore, there is an urgent need to develop the cost‐effective and sustainable rechargeable batteries. As a promising alternative, aqueous dual‐ion batteries (ADIBs) have attracted widespread attention due to their advantages of high safety, low cost, and non‐toxicity.^[^ ^4^, ^5^ ^]^ Currently, various of inorganic electrode materials including transition metal compounds, Prussian blue analogues, and poly anionic compounds have been achieved significant progress for boosting evolvement of the ADIBs.^[^ ^6^, ^7^ ^]^ However, most inorganic electrode materials with rigid structure are prone to severe volume changes, limited active site exposure and poor hydrophilicity, leading to electrode structure damage and capacity decay, thus affecting the cyclic stability and lifespan of the cells.^[^ ^8^, ^9^, ^10^ ^]^ In contrast, organic electrode materials possess flexible molecular construction and intrinsic hydrophilicity, availing to alleviate volume and interfacial effects. Besides, exposing large number of active functional groups enables them with high redox activity and excellent electrochemical reversibility.^[^ ^11^, ^12^, ^13^ ^]^ Therefore, organic electrode materials promise the advanced ADIBs.

The organic electrode materials store ions and charge through a unique coordination mechanism with charge‐compensating ions. Depending on the types of charge‐compensating ions, the organic electrode materials with redox functional groups can be classified as n‐, p‐, and bipolar‐type.^[^ ^14^, ^15^, ^16^, ^17^ ^]^ Among them, the bipolar organic electrode materials provoke tremendous interest because of their synergistic coordination with both anions and cations, which can be utilized as the symmetric electrodes to assemble the all‐organic ADIBs, obviating the limitations of employing two different electrode materials.^[^ ^18^ ^]^ Nevertheless, one issue is conscripted to concern that most of organic electrode materials with small molecule weight are suffering from an ease‐solubility, resulting in poor cycling stability.^[^ ^19^, ^20^, ^21^ ^]^ Furthermore, the majority of polymer materials exhibit poor electrical conductivity, served as one of the most limited factors, hindering the advancement of polymer‐based electrode materials. Covalent organic frameworks (COFs) are a class of crystalline framework materials with periodic array structures, high chemical stability, and essentially insoluble in the aqueous electrolytes. As well, their high specific surface area and abundant porous structure enable COFs with rapid ion‐transport capability. Specially, COFs with the adjustable imide radicals have gradually attracted more attention, because the presence of lone pair electrons in the imide groups improves their electrochemical activity.^[^ ^22^, ^23^ ^]^ Whereas, most of COFs with a single aperture structure cannot better accommodate the dual‐ion storage because of the selectivity of anions and cations with different radius to the pore sizes.^[^ ^24^ ^]^ Conversely, constructing COFs with bipolar functional groups and hierarchical pore structure containing both micropores and mesopores can offer dual active centers and transport pathways for synergistically anionic and cationic storage. Currently, bipolar COFs materials are mainly used in energy‐storage devices with organic electrolyte system, while their applications in aqueous batteries are limited because of constraints on the reversible redox reactions and the inability to achieve theoretical specific capacity.^[^ ^25^, ^26^ ^]^

Herein, this study presents the well‐design and construction of a polyimide‐based COF (labeled as NTPI‐COF) through the dehydration condensation reaction of two organic monomers including the n‐type 1,4,5,8‐naphthalenetetracarboxylic anhydride dianhydride (NTCDA) and the p‐type N,N,N′,N′‐Tetra(p‐aminophenyl)‐p‐phenylenediamine (TPPDA). The as‐fabricated NTPI‐COF featured with hierarchically porous structure and abundant redox active sites was enabled as the bipolar electrode because of the concomitant p‐type and n‐type functional groups, which is more favorable to utilize in the symmetric all‐organic ADIB. The electrochemical redox process and ion storage mechanism of the bipolar NTPI‐COF electrode in a 0.5 m NaCl aqueous solution were thoroughly investigated using in situ Raman, operational electrochemical impedance spectroscopy (EIS), and ex situ X‐ray photoelectron spectroscopy (XPS) detection. Results demonstrate that the synergistic effect of the redox groups (C═O and C─N) enables NTPI‐COF to exhibit dual‐redox activity and to achieve reversible coordination interactions with Na^+^ cations and Cl^–^ anions during the electrochemical reaction processes. As a result, the NTPI‐COF electrodes deliver a high reversible specific capacity of 109.7 mA h g^−1^ for Na^+^ storage and 74.8 mA h g^−1^ for Cl^–^ storage at a current density of 1 A g^−1^, respectively, and achieve a high capacity retention ratio of 93.2% over 10 000 cycles at 10 A g^−1^. In addition, the symmetric all‐organic ADIBs present an expanded average working voltage of 1.3 V, releasing an impressive energy density of 43 W h kg^−1^ at a power density of 2600 W kg^−1^. This work provides a pioneering strategy for designing bipolar organic electrodes and contributes to the development of advanced all‐organic ADIBs.

## 2 Results and Discussion

### 2.1 Synthesized Mechanism and Structure of NTPI‐COF

**Figure 1:**
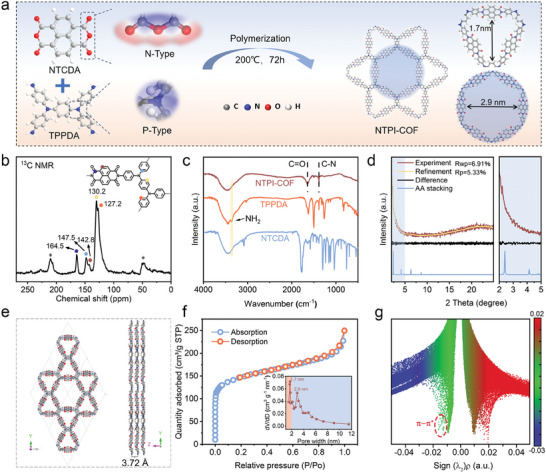
Synthetic route and structural analysis of NTPI‐COF. a) Schematic illustration of the synthetic process. b) NMR spectra. c) FTIR spectra. d) XRD patterns of the measured and simulated. e) Front and side view of the AA stacking model. f) N_2_ adsorption/desorption isotherm and pore size distribution (Inset). g) RDG plot.

**Figure** 1 illustrates the molecular structure and synthesis route of the NTPI‐COF, in which the two molecule monomers of NTCDA and TPPDA react through a dehydration condensation. According to the Schiff‐base reaction mechanism, the acid anhydride of NTCDA and the amino group of TPPDA can condense to form a series of covalent imine structures during the entire solvothermal reaction process.^[^ ^27^ ^]^ By continuous formation of amidation, the covalent imine structures are stacking to form a stable COF network. According to the molecular chain structures of the two monomers, the constructed COF subject theoretically possess non‐uniform pore sizes, which will be verified by the below pore structure analysis.

The chemical structure of NTPI‐COF was determined using ^13^C nuclear magnetic resonance (NMR) spectroscopy, Fourier transform infrared spectroscopy (FTIR), and Raman spectrum. In the solid‐state ^13^C NMR spectrum (Figure 1b), the signal peaks centered at 164.5 and 147.5 ppm correspond to carbonyl carbon atoms and aromatic carbon atoms adjacent to nitrogen atoms, respectively. Peaks at 130.2 and 127.2 ppm are attributed to other aromatic carbon atoms.^[^ ^28^ ^]^ Moreover, in the FTIR spectrum of NTPI‐COF (Figure 1c), a characteristic peak located at 1667 cm^−1^ corresponds to the carbonyl (C═O) group and another peak at 1340 cm^−1^ is assigned to the C─N─C chemical bond, indicating the formation of imine rings in the framework.^[^ ^29^ ^]^ Correspondingly, the original peak at 3356 cm^−1^ belong to the –NH_2_ group in TPPDA, is absent in the FTIR spectrum of NTPI‐COF.^[^ ^30^ ^]^ In addition, compared with the Raman spectra of the two monomers, an obvious C═O vibration peak arises at the Raman shift of 1715 cm^−1^ in the NTPI‐COF (Figure S1, Supporting Information). Such the above‐mentioned results comprehensively reveal the successful construction of the NTPI‐COF molecular structure configurated with abundant bipolar functional groups. According to the thermogravimetric analysis, NTPI‐COF can maintain a mass retention rate of up to 80% even over 500 °C (Figure S2, Supporting Information), demonstrating its excellent thermal stability, which is mainly attributed to its robust organic frameworks joined by the covalent bonds.

The small angle X‐ray diffraction (XRD) was performed to detect the crystallinity of NTPI‐COF (Figure 1d). The architectural models incorporating AA and AB stacking were constructed (Figure 1e; Figures S3 and S4, Supporting Information) and subsequently utilized to simulate theoretical XRD patterns. Significantly, a strong diffraction peak centered at 2.4° in the magnifying XRD pattern at small angle (Figure 1d) is assigned as the (100) lattice plane of NTPI‐COF, which is consistent with the simulated AA stacking model (Figure 1e), indicating the existence of an ordered structure in the constructed NTPI‐COF.^[^ ^31^ ^]^ To probe the pore structure of NTPI‐COF, the N_2_ absorption‐desorption isotherm was performed and exhibits a typical *IV*‐Type pattern as shown in Figure 1f. A sharp increase in the low‐pressure region of the absorption curve and a significant hysteresis loop in the medium‐pressure region of the absorption‐desorption curves indicate the coexistence of micropores and mesopores in the COF skeleton.^[^ ^32^ ^]^ The BJH pore size distribution curve exactly reveals the simultaneous presence of hierarchical pore structure with the average pore apertures of 1.7 and 2.9 nm in the NTPI‐COF matrix, which precisely matched with the simulated theoretical pore sizes (Figure 1a). As well, the BET specific surface area of NTPI‐COF is calculated up to 512 m^2^ g^−1^. The hierarchically porous structure and highly specific surface area of NTPI‐COF facilitate the insertion and transport for large‐sized cations and anions, and benefit to the full exposure of redox active sites, thereby enhancing the electrochemical activity.^[^ ^33^ ^]^ In addition, the localized orbital locator‐*π* (LOL‐*π*) color‐filled map (Figure S5, Supporting Information) displays high conjugation and good delocalization throughout the structural units of NTPI‐COF, which facilitates the enhancement of the electronic conductivity and charge transport capability.^[^ ^34^ ^]^ As displayed in Figure 1g, the reduced density gradient (RDG) and related gradient iso‐surface were conducted to identify the weak interactions among the skeleton networks in NTPI‐COF. It is worth noting that the green spike positioned at –0.02 to 0.00 *a.u*. of the sign (*λ*_2_)*ρ* in the RDG simulation (red dashed circle) represents the characteristic weak *π‐π* interaction between adjacent planar layers in the integral structure of NTPI‐COF. Such the *π‐π* overlap interaction ensures the high‐dynamic redox chemistry and efficient interface charge transfer in NTPI‐COF.^[^ ^35^, ^36^ ^]^

**Figure 2:**
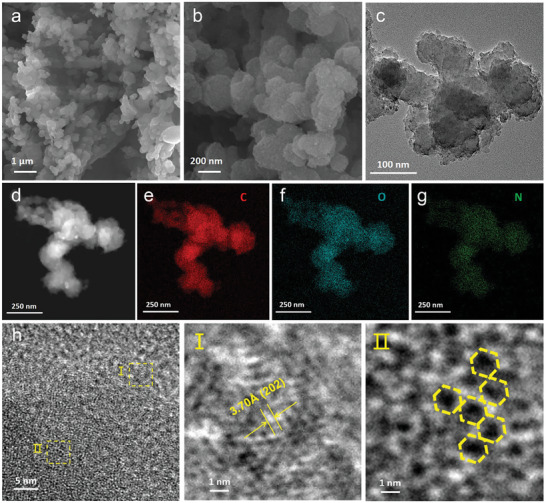
Morphology and microstructure of NTPI‐COF. a,b) SEM images. c) TEM image. d–g) Annular dark‐field TEM image and EDX mapping images. h) HRTEM image. Sections I and II are the magnifications of the labeled portion in h).

The morphology and microstructure of NTPI‐COF were probed by scanning electron microscopy (SEM) and transmission electron microscopy (TEM) observations. SEM images of NTPI‐COF reveal a nano‐spherical morphology with an average grain size of 100 nm (**Figure** **2**a,b), which is further verified by TEM imaging (Figure 2c). The distribution of elements is further disclosed through the energy‐dispersive X‐ray spectroscopy (EDX) mapping, demonstrating the uniform distribution of carbon (C), nitrogen (N), and oxygen (O) elements within the spherical NTPI‐COF matrix (Figure 2d–g). High‐resolution transmission electron microscope (HRTEM) observations unveil a partially ordered structure and porous microstructure features of NTPI‐COF (Figure 2h). Although the entire area is predominantly amorphous, a certain of localized regions show weak crystalline tendencies, which echoes the unconspicuous diffraction peaks detected in the XRD pattern. For all this, it is noteworthy that a long‐range disordered structure can facilitate ionic migration in NTPI‐COF, as well as the short‐range ordered structures can mitigate the blocking effect of dense crystal lattice to enhance the charge transport capability.^[^ ^37^ ^]^ Additionally, the magnified section I tagged in Figure 2h reveals closely ordered structures in NTPI‐COF matrix, measured an interplanar spacing of 3.70 Å, which is matched well with the theoretical simulation value of 3.72 Å in the AA stacking NTPI‐COF model (Figure 1e). While in the magnified section II, HRTEM image also illustrates the irregular hexagonal pore structure, which is consistent with the molecular configuration of NTPI‐COF. Such the above results further provide powerful evidence for revealing the successful construction of the well‐designed NTPI‐COF.

### 2.2 Na^+^ Storage Behavior of NTPI‐COF

**Figure 3:**
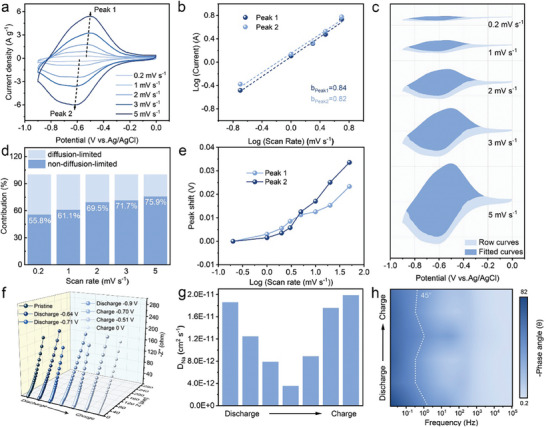
Quantitative electrochemical kinetics analysis of NTPI‐COF for Na^+^ storage. a) CV curves at different scan rates. b) The linear relation of logarithm dependence between peak current densities and scan rates. c) Voltammetric response at different scan rates. d) The proportion of diffusion‐ and capacitive‐controlled capacity contribution at different scan rates. e) Peak potential shifts between the cathodic/anodic peaks with the scan rates ranging from 0.2 to 50 mV s^−1^. f) EIS plots and g) the corresponding range of the Na^+^ diffusion coefficients at different potentials. h) Bode plots during charging and discharging.

In a 0.5 m NaCl solution electrolyte, the electrochemical performance of NTPI‐COF electrode for Na^+^ storage in a half‐cell was investigated using a typical three‐electrode testing system with an optimized cut‐off voltage of –0.9–0 V. First, the obtained cyclic voltammetry (CV) curves (**Figure** **3**a) show a pair of symmetrical redox peaks, indicating the presence of available redox‐active sites within the NTPI‐COF electrode for effective Na^+^ uptake and removal. At the higher scan rates, the reduction peaks slightly shift to the negative direction while the oxidation peaks shift equivalently in the positive direction. Only a minimal potential shift appears among the cathodic/anodic peaks, implying that the NTPI‐COF electrode possesses favorable redox kinetics.^[^ ^38^ ^]^ Furthermore, the peak current (*i_p_*) exhibits almost linear growth with an increase of scan rates (Figure S6, Supporting Information). All of the calculated *b* values, according to the equation: *i_p_* = *av^b^*, where *i_p_* represent the peak current values and *v* represent the scan rates, are close to 1.0 (Figure 3b).^[^ ^39^ ^]^ This further indicates that the NTPI‐COF electrode with the feature of ultrafast redox kinetics property works through a charge storage mechanism dominated by capacitive control. The capacitive contribution of the NTPI‐COF electrode is quantified in Figure 3c according to the equation: *i* = *k_1_v* + *k_2_v^1/2^*, where *k_1_v* and *k_2_v^1/2^* represent the surface reaction‐dominated process and the diffusion‐dominated process at the specified potential, respectively.^[^ ^40^ ^]^ The capacitive‐controlled capacity contribution exhibits an increasing trend as the scan rates continuously increase, ranging from 55.8% at 0.2 mV s^−1^ to 75.9% at 5 mV s^−1^ (Figure 3d), which indicates that the pseudocapacitive‐controlled electrochemical reaction process predominates at high charge‐discharge rates. In addition, when the scan rates range from 0.2–50 mV s^−1^, all cathodic/anodic peaks show only small peak potential shifts (<0.05 V) (Figure 3e; Figure S7, Supporting Information), which serves as an indicator that the pseudocapacitive‐controlled Na^+^ storage behavior favors rapid redox kinetics.^[^ ^41^ ^]^

To further verify the rapid redox kinetics, the operational electrochemical impedance spectroscopy (EIS) analysis was conducted on the NTPI‐COF electrode, and the fitted equivalent electrical circuit (EEC) models (Figure S8, Supporting Information) provide the quantitative analysis of the EIS data. It is evident that all of the Nyquist plots at real‐time potentials (Figure 3f) exhibit almost minimal differences. The charge transfer resistances (R_ct_) at the interface among the NTPI‐COF electrode and electrolyte, reflected by the diameter of the semicircle in high‐frequency region, remain extremely consistent along with Na^+^ uptake/removal (Figure S9, Supporting Information). Furthermore, the calculated Na^+^ diffusion coefficient (D_Na_^+^) of the NTPI‐COF electrode (Figure S10, Supporting Information) reveals that the values of D_Na_^+^ exhibit a trend of initial decrease followed by an increase (Figure 3g). Such the phenomenon arises from the insertion of Na^+^ ions into COF during the discharging process, leading to the occupation of active sites and an increase in spatial hindrance as well as restricted ion diffusion. Conversely, during charging process, Na^+^ ions gradually depart, resulting in a reduction of spatial hindrance and the restoration of rapid ion migration capability.^[^ ^27^ ^]^ Throughout the whole electrochemical process, the high values of D_Na_^+^ (>10^−12^ cm^2^ s^−1^) persist, primarily due to the hierarchical porous structure of NTPI‐COF facilitating the rapid ion diffusion. This indicates the excellent electrochemical kinetic characteristics of the NTPI‐COF electrode, allowing for rapid Na^+^ migration and storage.^[^ ^42^ ^]^ In addition, in the Bode plot (Figure 3h), the dashed line at a phase angle of 45° corresponds to the characteristic frequency (*f*_0_) ranging from 0.33 to 1.72 Hz, which means the ultra‐fast frequency response (τ_0_) only with a minimum interval of 0.58 s, further suggesting that the NTPI‐COF electrode possesses rapid redox kinetics and fast electrochemical response.^[^ ^43^ ^]^

**Figure 4:**
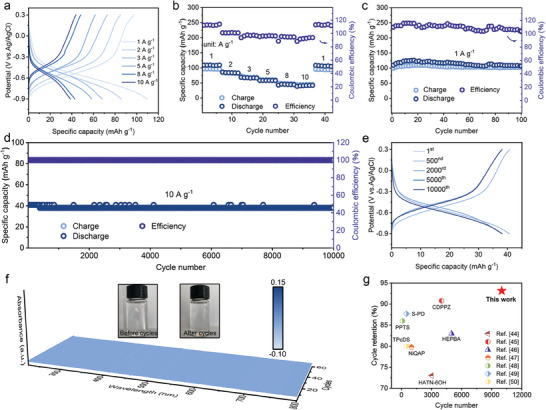
Na^+^ storage capability of NTPI‐COF for ADIB. a) Charging‐discharging curves at various rates. b) Rate capacity. c) Cycle performance at 1 A g^−1^ for 100 cycles. d) Long‐term cycling stability at 10 A g^−1^ over 10000 cycles. e) GCD curves at varying cycle indexed in long‐term cycling. f) Corresponding in situ UV–vis spectroscopy of electrolyte during the charging‐discharging process. The insets show the electrolyte images before and after cycling. g) The compared cycling stability with the reported electrodes for Na^+^ storage.

**Figure** **4**a shows the charge‐discharge curves of the NTPI‐COF electrode for ADIB anode at different current densities. During the charge‐discharge process, the voltage platforms of these curves are consistent with the oxidation‐reduction peaks in the CV curves. At the rates of 1, 2, 3, 5, 8, and 10 A g^−1^, NTPI‐COF exhibits high discharge specific capacities of 109.7, 85.7, 68.2, 58.3, 42.8, and 38.7 mA h g^−1^, respectively. The rate performance chart (Figure 4b) shows that the capacity can be restored to 108.1 mA h g^−1^ when the rate drops back to 1 A g^−1^. At the lower current densities, the values of Coulombic efficiency (CE) exceed 100%, yet with an increase of current density, the CE gradually decreases. Figure 4c illustrates the galvanostatic charge‐discharge performance of NTPI‐COF at 1 A g^−1^, demonstrating a high reversible capacity of 110.2 mA h g^−1^ and a capacity retention of 97.8% after 100 cycles. What's exciting is that the NTPI‐COF electrode can deliver an reversible specific capacity of 40.9 mA h g^−1^ under a rate of 10 A g^−1^, with a capacity retention rate of 93.2% over 10000 cycles (Figure 4d). Meanwhile, the CE is close to 100% throughout the cycle process, indicating the excellent long‐term cycling stability and reversibility. Furthermore, during the cyclic process, no significant deviation is observed in the voltage platforms indicated by the charge‐discharge curves at varying cycle indexes (Figure 4e). In situ UV–vis spectroscopy was further performed to investigate the electrolyte condition during charge‐discharge process of the NTPI‐COF electrode. As shown in Figure 4f, there are no significant absorption peak or color change in the electrolyte after repeated cycling, indicating the robust structural stability of NTPI‐COF in the aqueous electrolyte. In contrast, both the NTCDA monomer and TPPDA monomer electrodes display varying degrees of capacity decay after short‐time cycling at 10 A g^−1^ (Figure S11, Supporting Information), and the color of the electrolyte changes significantly, indicating that the constructed COF matrix after polymerization can effectively suppress monomer dissolution. Cycling stability of NTPI‐COF outperforms previous results of organic electrodes for Na^+^ storage reported in the literatures (Figure 4g).^[^ ^44^, ^45^, ^46^, ^47^, ^48^, ^49^, ^50^ ^]^ Therefore, NTPI‐COF electrode with excellent structural characteristics possesses great potential in the application of ADIBs anode for Na^+^ storage.

### 2.3 Electrochemical Performance of the All‐Organic Symmetric ADIB

**Figure 5:**
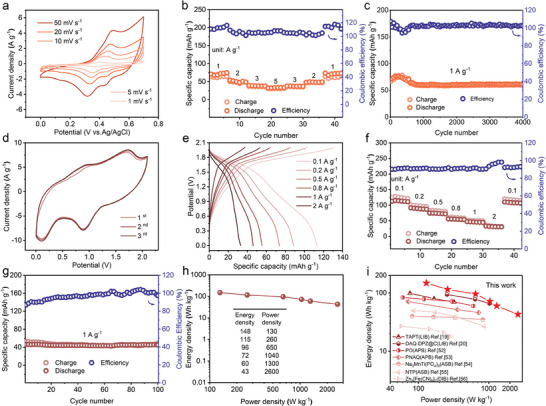
Cl^−^ storage behavior of NTPI‐COF and the electrochemical performance of the symmetric full‐cell. a–c) CV curves, rate capacity, and cycle performance of the NTPI‐COF electrode for Cl^−^ storage. d–g) CV curves, charge‐discharge curves, rate capacity, and cycle performance of the full‐cell. h) Energy density and power density profile. i) Energy density comparison with the reported symmetric full‐cells.

Due to the polyimide‐based COF cathode through the adsorption of electron‐accepting anions for energy storage,^[^ ^28^ ^]^ the application of NTPI‐COF for Cl^–^ ion storage as the ADIB cathode was further investigated. As shown in **Figure** **5**a, the CV curves of the NTPI‐COF cathode exhibit a distinct pair of oxidation and reduction peaks in the cut‐off potential range of 0–0.7 V, indicating the presence of active redox sites during the cathodic reaction. With the increase of scan rates, the peak shape of CV curves remains almost unchanged, while the polarization potentials between the oxidation and reduction peaks only slightly increase, demonstrating a high oxidative‐reductive reversibility of the NTPI‐COF even under the superfast charge‐discharge conditions. The capacitance contribution (Figure S12, Supporting Information) and *b*‐value profiles (Figure S13, Supporting Information) showcase that the electrochemical reaction process of the NTPI‐COF is mainly diffusion‐controlled. The charge‐discharge curves at different current densities exhibit distinct discharge plateaus (Figure S14, Supporting Information), which is consistent with the preceding CV findings. Figure 5b displays the rate capacity of the NTPI‐COF cathode. The specific capacity remains stable at 74.8, 48.9, 37.9, and 33.3 mA h g^−1^ when the rates increase from 1.0 to 5.0 A g^−1^, demonstrating good rate capacity retention. At lower current densities, such as 1.0 A g^−1^, NTPI‐COF initially exhibits a CE of 110%, gradually stabilizing at 100% as the current density increases. After charge‐discharge cycling for 4000 times at 1 A g^−1^ (Figure 5c), a reversible capacity of 61.4 mA h g^−1^ can be maintained, with a cyclic capacity retention of 86.4%, indicating significant redox reversibility and structural stability. The results described above demonstrate that the NTPI‐COF electrode concurrently served as a cathode for ADIBs can still exhibit high capacity, good rate capability, and cycling stability.

As a result, considering the bipolar characteristics of NTPI‐COF, the electrochemical performance of the all‐organic symmetric ADIB (full‐cell) was further evaluated within the optimized cut‐off voltage range of 0–2 V. The mass loading of active materials was matched based on the discharge specific capacities of NTPI‐COF in the cathode (for Cl^–^ storage) and anode (for Na^+^ storage) gained in their corresponding half‐cells. According to the equation: *m_1_Q_1_* = *m_2_Q_2_*, where *m* stands for active mass and *Q* is specific capacity, the calculated mass of the active materials loading in cathode and anode of the full‐cell are 1 mg cm^−2^ and 0.66 mg cm^−2^, respectively. Under an open current voltage of 0.06 V, CV curves of the full‐cell are collected at a scan rate of 50 mV s^−1^ for the first three cycles. As displayed in Figure 5d, two obvious oxidation peaks appear at 1.0 and 1.7 V, while the reduction peaks locate at 0.2 and 0.9 V, which are almost well‐matched with the CV results presented in their corresponding half‐cells. Charge‐discharge curves of the full‐cell, as shown in Figure 5e, obviously exhibit consistent voltage platform and overpotential at different current densities, implying that the NTPI‐COF electrode possesses good rate capability when served as the symmetric electrodes.^[^ ^51^ ^]^ Consequently, the full‐cell delivers an initial discharge specific capacity of up to 112.7 mA h g^−1^ at 0.1 A g^−1^, and a reversible capacity of 47.1 mA h g^−1^ can be achieved at 1 A g^−1^ (based on the mass loading of the active material in anode), indicating its excellent rate performance and high reversibility (Figure 5f). Charge‐discharge cycle performance of the full‐cell was performed at 1 A g^−1^ (Figure 5g). It's worth noting that CE of the full‐cell does not yet reach to 100% at the beginning of charge‐discharge cycles, which mainly because the symmetric organic electrodes are not fully activated so that the active sites are underutilized at the outset, specially under a large current density. Whereas, after charging‐discharging for dozens of cycles, the full‐cell delivers a high reversible capacity of 45.3 mA h g^−1^ with a capacity retention rate of 98.3% and a CE approach 100%, demonstrating a finished activation process and an outstanding cycle stability. More notably, the full‐cell with an average operating voltage of 1.3 V (which is defined as the effective integral area of the charge and discharge curves divided by the specific capacity) exhibits the energy densities of up to 148 and 43 W h kg^−1^ at the power densities of 130 and 2600 W kg^−1^ (Figure 5h), respectively, surpassing the most reported symmetrical full‐cells to date (Figure 5i).^[^ ^19^, ^20^, ^52^, ^53^, ^54^, ^55^, ^56^ ^]^

### 2.4 Storage Mechanism of NTPI‐COF for the Symmetric ADIB

**Figure 6:**
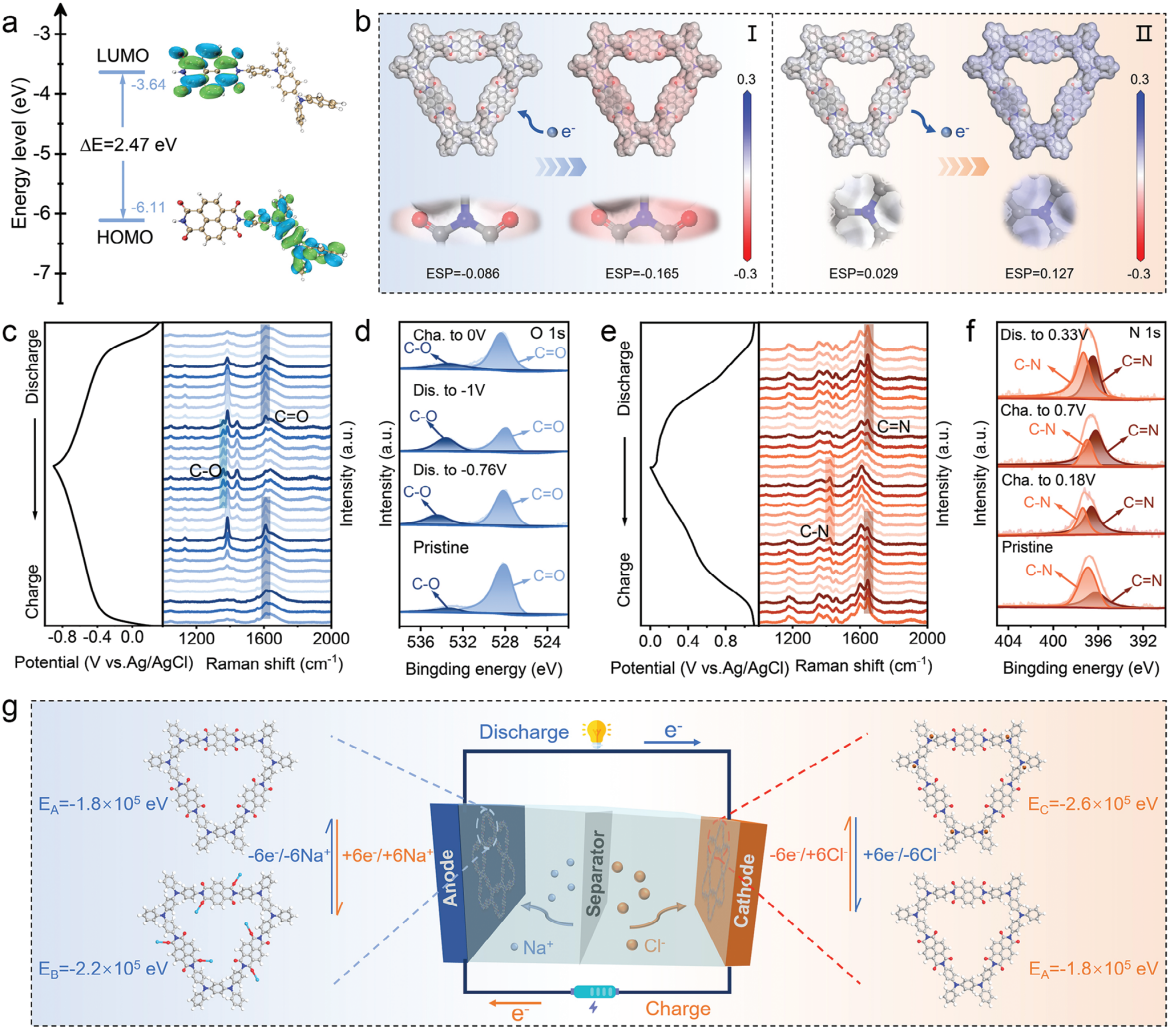
Charge/ion storage mechanism of NTPI‐COF as the symmetric electrodes for full‐cell. a) HOMO‐LUMO energy levels. b) ESP of a molecular repeating unit (at the initial state and after injecting/extracting electrons). c–f) In situ Raman and ex situ XPS spectra of the NTPI‐COF electrodes during the discharge/charge process. g) Schematic illustration of the working symmetric full‐cell and structural evolution of the NTPI‐COF molecules upon Na^+^ and Cl^–^ uptake/removal.

To reveal the intrinsic mechanism of NTPI‐COF as the symmetric electrodes for ADIB, the ion/charge storage behaviors were investigated by theoretical calculations and in situ characterizations. First, the lowest unoccupied molecular orbital (LUMO) and highest occupied molecular orbital (HOMO) energy levels were calculated to probe the electronic structure of NTPI‐COF (**Figure** **6**a). On one hand, NTPI‐COF exhibits a low LUMO energy level of –3.64 eV, which is attributed to the hierarchical porous structure improved the *π*‐orbital electron delocalization effect and thus enhances the electron affinity. Furthermore, a small energy level gap (*ΔE*) of 2.47 eV between LUMO and HOMO demonstrates the good electronic conductivity of NTPI‐COF, which is one of the main reasons why the symmetric full‐cell delivers a good rate capability and high energy density.^[^ ^57^ ^]^ Besides, as illustrated in Figure S15 (Supporting Information), a comparison of the *ΔE* values was conducted among NTPI‐COF and some typical organic electrode materials reported previously. It is found that NTPI‐COF possesses the lowest LUMO value and the smallest *ΔE*, which is mainly owing to the continuous skeleton structure of the polymeric COF compound.^[^ ^40^ ^]^ In order to investigate the charge storage behavior, the molecular electrostatic potentials (ESPs) of a repeating unit in NTPI‐COF were probed after injecting and extracting electrons (Figure 6b).^[^ ^58^ ^]^ In the scenarios, involving the repeating units with and without electrons, the O atom positions in imine group exhibit the lowest ESP, indicating their nucleophilic nature towards binding cations.^[^ ^59^ ^]^ Meanwhile, the N atom locations in TPPDA moiety display electrophilicity, with the highest ESP near the C─N bond upon electron extraction, suggesting it as the active site for binding anions.

To further identify the electrochemical active centers of NTPI‐COF, in situ Raman spectroscopy was performed during the anodic and cathodic reaction process. Prior to the discharging, Raman spectra of the NTPI‐COF anode (Figure 6c) show a regular change of the vibration peak centered at 1620 cm^−1^, corresponding to the C═O bond of imine group in the active unit of NTPI‐COF. As the discharge process intensified, the peak intensity of the C═O bond (deep blue‐shaded region) significantly diminishes, while new peaks arise concomitantly at 1380 cm^−1^, which are related to the formation of C─O─Na bonds (light blue‐shaded region). Namely, the reduction of C═O bonds causes cationic coordination through aldolization, forming C─O─Na bonds upon embedding Na^+^ ions and accepting electrons. With the potential increasing, the signal of the C─O─Na bond gradually disappears as the C═O bond generated during the oxidation reaction. After removing Na^+^ ions completely, the C═O bonds revive as before, confirming the high redox reversibility of the carbonyl substituent. Additionally, in situ Raman spectra in the cathodic reaction show that the vibration peak intensity of the C─N bonds at 1420 cm^−1^ (orange‐shaded region) weakens when charging and intensifies gradually when discharging (Figure 6e). Simultaneously, the vibration peak of C═N─Cl bond at 1650 cm^−1^ (deep brown‐shaded region) first presents and then absents during charging and discharging process, inferring that the C─N centers are the active sites for Cl^–^ uptake.^[^ ^44^, ^60^ ^]^

Besides, ex situ XPS spectra of the electrode surface were conducted for the two NTPI‐COF electrodes. On the anodic side, the relative peak area of C═O bond assigned in the O1s XPS spectrum gradually decreases as the discharge deepened, while that of C─O bond gradually increases (Figure 6d). When the voltage returned to 0 V, the peak areas of C═O and C─O bond return to their initial states. Remarkably, the C═O bonds are not fully utilized when the NTPI‐COF anode proceeds the redox reaction, because there is a repulsive force among adjacent charges, ion embedding is a stepwise reaction process, and the utilization of carbonyl groups at different positions requires the assigned voltage windows. Within the voltage window range of 0–2 V, each structural unit can only utilize three pairs of carbonyl groups.^[^ ^61^ ^]^ Similarly, during the charging process of the NTPI‐COF cathode, the peak area of C─N bond in the N1s XPS spectrum gradually decreases, while that of C═N bond increases (Figure 6f). When discharged to 0.33 V, the peak area of C─N exceeds that of C═N, which is consistent with the in situ Raman results (Figure 6e). Schematic illustration of the working mechanism for the symmetric NTPI‐COF//NTPI‐COF full‐cell is shown in Figure 6g. Coordination pathways of Na^+^ and Cl^–^ ions during charge‐discharge process were computationally simulated using Density Functional Theory (DFT). According to the calculated binding energy of Na^+^ and Cl^–^ ions coordinated with bipolar NTPI‐COF (Figure 6g; Figure S16, Supporting Information), three pairs of diagonal C═O positions of NTPI‐COF are more readily to bind the Na^+^ ions and accept electrodes as depicted on the anode side, while six C─N active centers are inclined to uptake Cl^–^ ions and release electrodes in the cathode. As a result, the above comprehensive analyses further prove the great potential of NTPI‐COF served as the bipolar electrodes for all‐organic symmetric ADIBs.

## 3 Conclusion

In summary, a structurally stable and hierarchically porous polyimide‐based covalent organic framework (NTPI‐COF) was fabricated by the dehydration condensation based on Schiff‐base reaction. The theoretical calculations and in situ/ex situ investigations reveal that NTPI‐COF with the feature of bipolarity provides reversible dual‐redox active centers for synergistic storage of cations and anions. Furthermore, the hierarchical pore structure and high electronic conductivity endow NTPI‐COF to facilitate rapid ion diffusion and charge transfer. In addition, the robust framework structure of NTPI‐COF ensures the reversible redox properties for ADIB electrode. As a results, the NTPI‐COF electrode delivers a high reversible capacity of 109.7 and 74.8 mA h g^−1^ at 1 A g^−1^ as the anode and cathode, respectively, and remain a high capacity retention of 93.2% over 10 000 cycles at 10 A g^−1^. As well, the all‐organic symmetric ADIB (full‐cell) with an average operating voltage of 1.3 V delivers a remarkable energy density of 148 W h kg^−1^ and a high power density of 2600 W kg^−1^. The proposed bipolar polyimide‐based COF, containing both n‐type and p‐type active centers, opens up an effective pathway for designing low‐cost and high‐performance all‐organic electrodes for symmetric ADIBs.

## Conflict of Interest

The authors declare no conflict of interest.

## Supporting information

Supporting Information

## Data Availability

The data that support the findings of this study are available from the corresponding author upon reasonable request.
