# Supplementary material for: Regulating the Porosity and Bipolarity of Polyimide‐Based Covalent Organic Framework for Advanced Aqueous Dual‐Ion Symmetric Batteries
Source: Adv Sci (Weinh). 2024 Aug 19;11(39):2407073. doi: 10.1002/advs.202407073 (PMC11496998; doi:10.1002/advs.202407073)
Supplement: Supplementary file 1 — Supporting Information [file ADVS-11-2407073-s001.docx]

***Supporting Information***

Regulating the Porosity and Bipolarity of Polyimide-based Covalent Organic Framework for Advanced Aqueous Dual-ion Symmetric Batteries

Dongxiang Geng*^1, #^*, Heng Zhang*^1, 2, #,^ **, Zhijian Fu*^1^*, Ziming Liu*^1^*, Yafei An*^1^*, Jun Yang*^1^*, Dawei Sha*^3^*, Long Pan*^4,^* *, Chao Yan*^1,^* *, ZhengMing Sun*^4^*

*1 School of Materials Science and Engineering, Jiangsu University of Science and Technology, Zhenjiang 212100, P R China;*

*2 Key Laboratory of Advanced Energy Materials Chemistry (Ministry of Education), Nankai University, Tianjin 300071, P R China;*

*3 Institute of Technology for Carbon Neutralization, Yangzhou University, Yangzhou 225009, P R China;*

*4 School of Materials Science and Engineering, Southeast University, Nanjing 210089, P R China*

*# These authors contributed equally to this work.*

** Corresponding authors:*

*chaoyan@just.edu.cn (C. Yan), hengzhang@just.edu.cn (H. Zhang),*

*panlong@seu.edu.cn (L. Pan)*

**Experimental Section**

***Chemical reagents.*** 1,4,5,8-naphthalenetetracarboxylic anhydride (NTCDA, 96%), N,N,N',N'-Tetra(p-aminophenyl)-p-phenylenediamine (TPPDA, 98%), Sodium chloride (99.5%), N-methylpyrrolidone (NMP, 99.5%), 1,3,5-Trimethylbenzene (97.0%), Isoquinoline (97.0%), N, N-dimethylformamide (DMF, 99.5%), polyvinylidene fluoride (PVDF, average Mw ~400000) and ethanol were purchased from Sigma. The above chemical reagents are not further purified.

***Synthesis of NTPI-COF.*** Schiff-base reaction was employed to synthesize the NTPI-COF. Firstly, NTCDA (0.27 g, 1 mmol) and H_2_O (5 mL) were added into 10 mL of NMP and sonicating for 20 min. Meanwhile, TPPDA (0.27 g, 0.5 mmol) was dissolved in 10 mL of NMP, then mixed with the above solutions and add 10mL 1, 3, 5-Trimethylbenzene and 0.5mL isoquinoline to the mixed solution, sonicating for 30 min. The above mixture was transferred to a high-pressure reaction kettle (NSG100-P5, 20 MPa, CHEM^N^) and reacted for 72 hours at 200 °C. Afterwards, a brown-red solid powder was obtained after washing with DMF, acetone and H_2_O in turn.

***Electrochemical measurements.*** Firstly, the as-prepared NTPI-COF powder, acetylene black, and PVDF were dispersed in NMP at a mass ratio of 7:2:1 to form a uniform slurry, which was then coated on carbon paper. The coated slurry was vacuum dried at 60°C for 12 hours, with the mass of active material coated on the carbon paper during the half-cell test was 0.91 mg. 0.5M NaCl aqueous solution was used as the electrolyte for half-cell and full-cell. Cyclic voltammetry (CV) and electrochemical impedance spectroscopy (EIS) measurements were implemented by using a multifunctional electrochemical workstation (Biologic SP-150). CV curves were collected under different scanning rates. EIS measurements were performed in a frequency range of 0.01–100 kHz with a voltage amplitude of 5 mV at different potentials during charge-discharge process. Galvanostatic charge-discharge (GCD) tests were conducted on a LAND-CT2001C tester (Wuhan LANHE) under different current densities. All of the electrochemical measurements were carried out at 25 °C.

***Characterization methods.*** Several characterization techniques were carried out for evaluating morphology, microstructure, molecular and chemical structures of the synthesized samples, including scanning electron microscope (SEM, FEI Nova NanoSem450), transmission electron microscope (TEM; Tecnai G220S-Twin), nuclear magnetic resonance spectra (NMR; Bruker Avance III spectrometer, 400 MHz), X-ray photoelectron spectroscopy (XPS; Al Kα Thermo electron), fourier infrared spectroscopy (FT-IR; Thermo Scientific Nicolet 6700), mass spectrometry (MS; Bruker rapiflex MALDI Tissuetyper), ultraviolet-visible spectrophotometer (UV-vis; Shanghai Metash Instruments Co., Ltd UV-8000), laser micro-Raman spectrometer (Raman; RENISHAW inVia), as well as X-ray diffraction (XRD; Bruker D8 X-ray spectrometer) equipped with a 2D detector (Cu Kα, λ=1.54 Å). Thermal gravimetric analysis (TGA; TGA5500) were performed under an inert atmosphere at arising speed of 5°C min^–1^. Nitrogen adsorption-desorption isotherm was investigated by a Quanta Autosorb-IQ2 instrument at 77 K. The total specific surface area was calculated by Brunauer-Emmet-Teller (BET) method.

***Theoretical calculations.*** The structure modeling was performed using Materials Studio (MS) software. Monomer fragments were linked together to generate a monolayer model, which was then filled into the unit cell with cell parameters of *a*=*b*=43.04 Å and *c*=3.72 Å (*α*=*β*=90°, *γ*=120°) and a symmetry group of P6 (C6-1) type. The overlapping stacking arrangements of AA and AB were constructed based on the aforementioned unit cell parameters, and geometry optimization was conducted using the DMol3 module. XRD spectra simulations were performed for both AA and AB stacking. Following that, the DMol3 module in the MS software was employed to theoretically calculate and analyze the electrostatic potential and binding energy of the NTPI-COF structure unit using density functional theory (DFT) method.

The calculations of HOMO-LUMO energy levels, RDG plot, and LOL-π map were conducted using the DFT method implemented in the commercial Gaussian 16 program package. The structures were fully optimized at the B3LYP-D3/6-311G(d) level of theory. The LOL-π and RDG wave function cubes were obtained using the Multiwfn 3.8 (dev) code and their visualization was achieved using the VMD software.^[1-3]^

The theoretical capacity (C_t_) of NTPI-COF is calculated based on the following Equation ^[4]^:

$$C_{t}=\frac{N_{e}\times96485}{3600\times M_{w}}\times1000$$

Where *N_e_* is the number of transferred electrodes, and *M_w_* is the molecular weight. The *M_w_* of the repeated unit of NTPI-COF is 1441.35 g·mol^-1^, and the number of active sites in each NTPI-COF repeated unit is 12, corresponding to 6 Na^+^ and 6 Cl^–^ ions for coordination and 12 electrodes for transference. Therefore, the theoretical capacities of both the cathode and the anode in NTPI-COF are equivalently 111.6 mA h g^-1^.

***In-situ electrochemical investigations.*** In situ Raman investigation was applied by DXR Raman microscope at the 532 nm excitation laser, wherein the laser power was set at 10 mW with a spot size of approximately 2.5 μm. The spectra were collected in scattering mode with 3 scans at 10 s each exposure time within the range from 1000 to 1800 cm^–1^. In situ Raman spectra of NTPI-COF electrode was collected from in-operando monitoring mode with a series of repetitive scans every 30 s during the CV electrochemical testing at a scan rate of 1 mV s^–1^. In order to collect the in situ UV-vis spectra during the charge and discharge process, an optical glass electrochemical tank filled with 0.5 M NaCl electrolyte was used as a reference cuvette. The NTPI-COF electrodes were put into the working cuvette for in situ electrochemical UV-vis measurements at a current density of 1 mA cm^–2^.

**Supporting Figures**


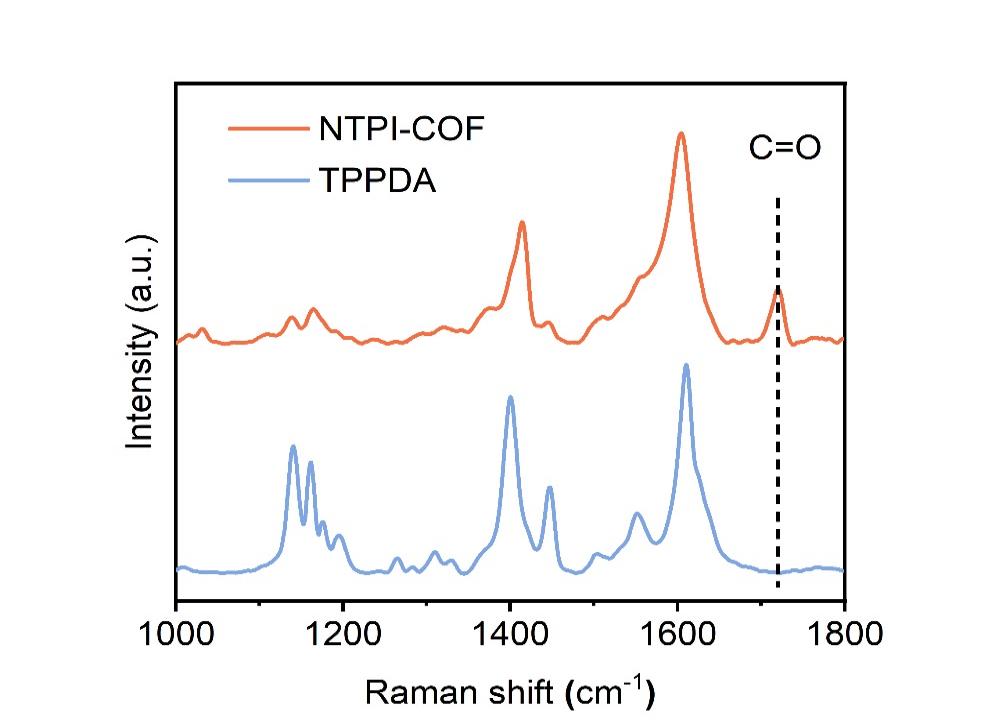


**Fig. S1.** Raman spectra of TPPDA monomer and NTPI-COF.


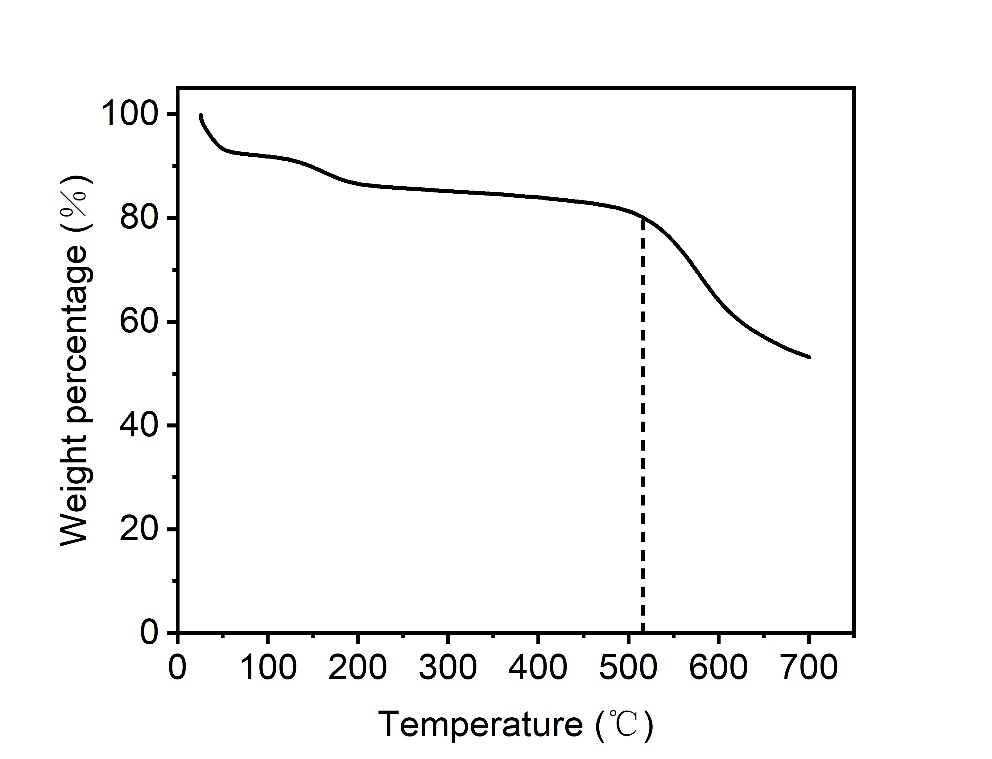


**Fig. S2.** TGA curves of NTPI-COF.


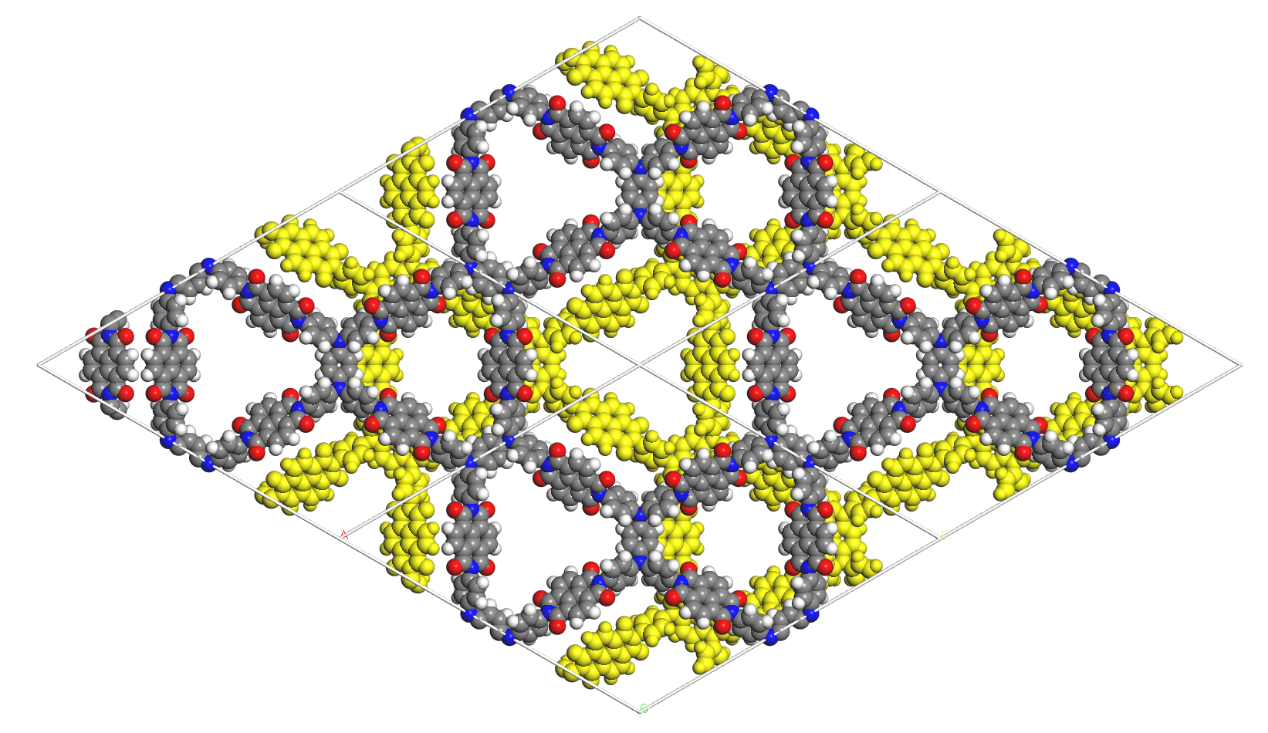


**Fig. S3.** The AB stacking model of the hexagonal structure of NTPI-COF.


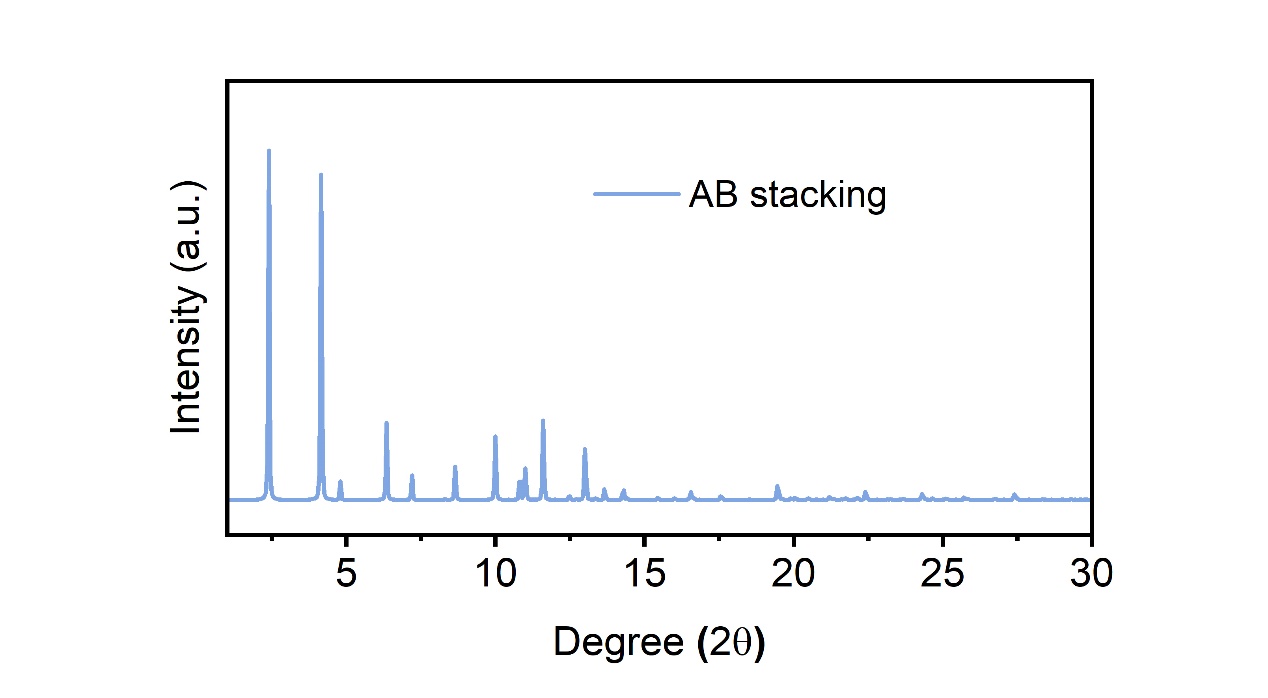


**Fig. S4.** Simulated XRD pattern of AB stacking model.


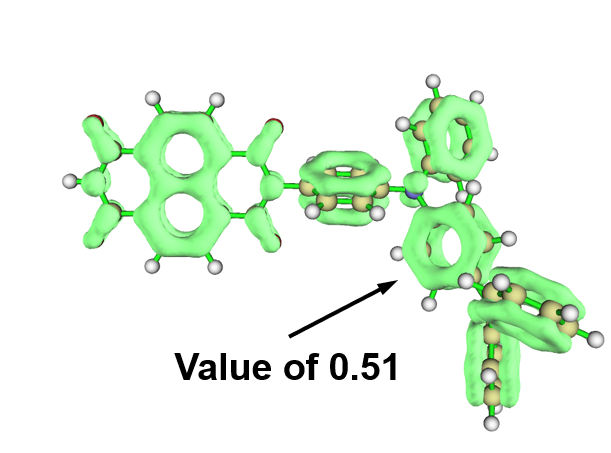


**Fig. S5.** LOL-π map of NTPI-COF.


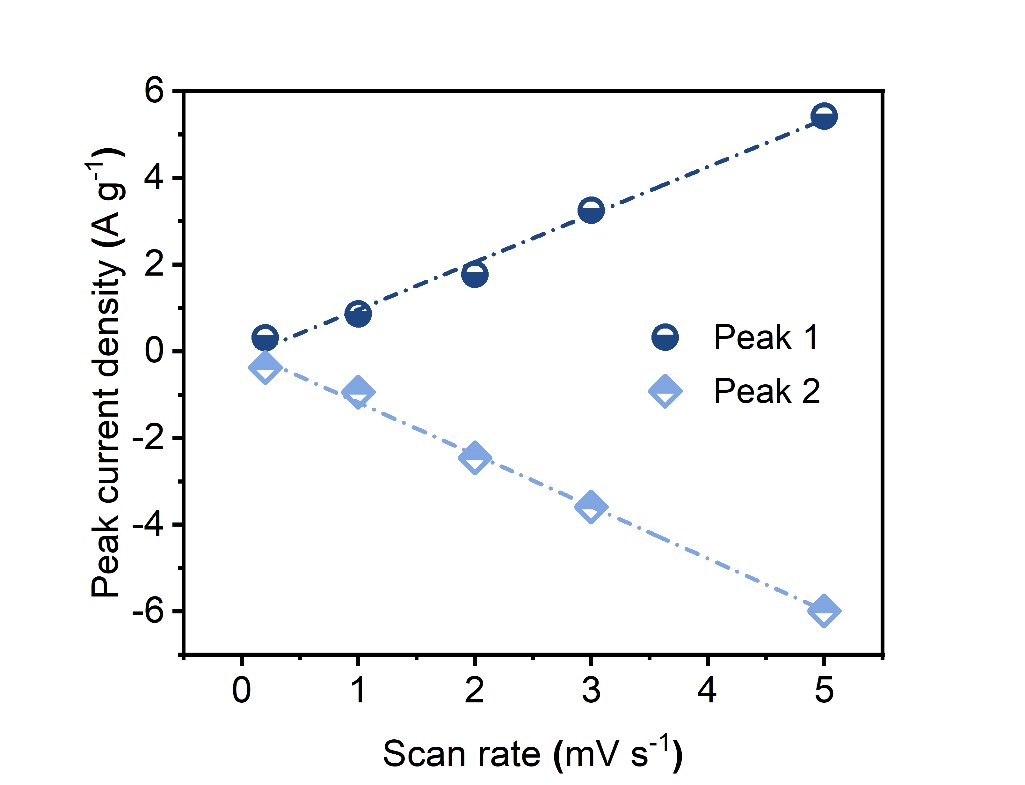


**Fig. S6.** Linear fits of cathodic and anodic redox peak currents.


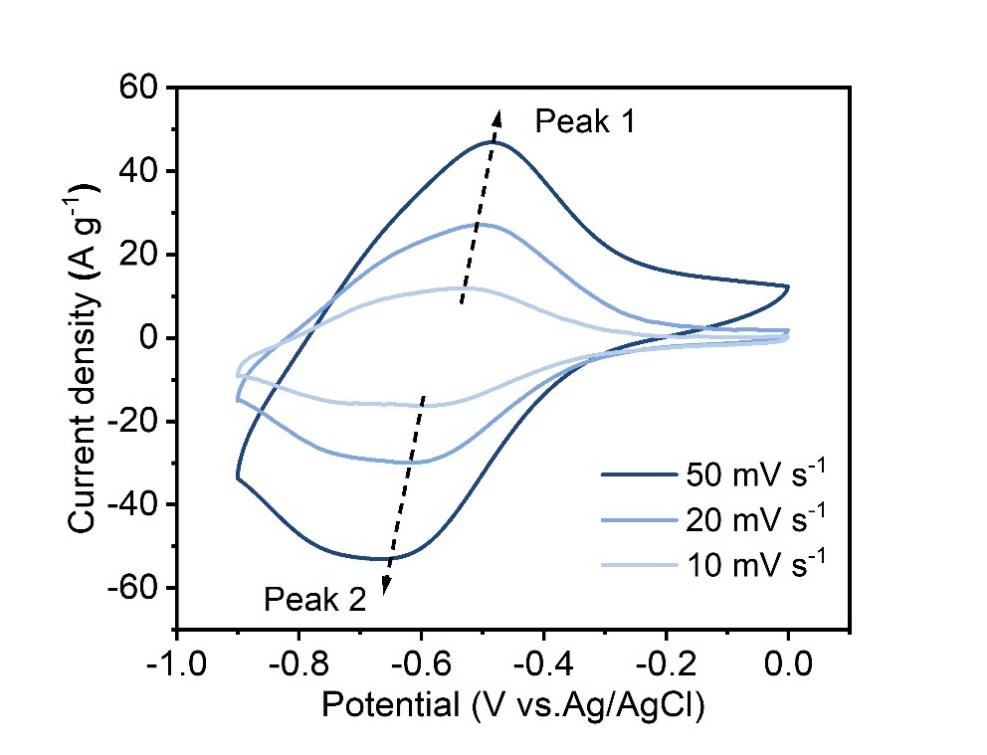


**Fig. S7.** CV curves at multiple scan rates from 10 to 50 mV s^–1^.


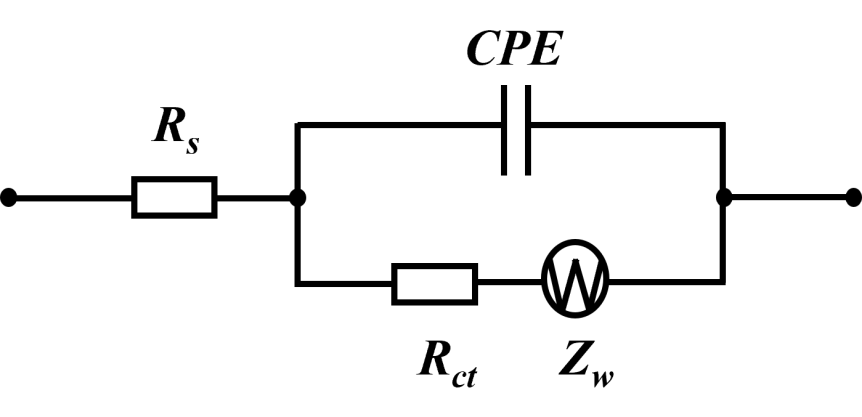


**Fig. S8.** Electrical equivalent circuit models for fitting the EIS data.


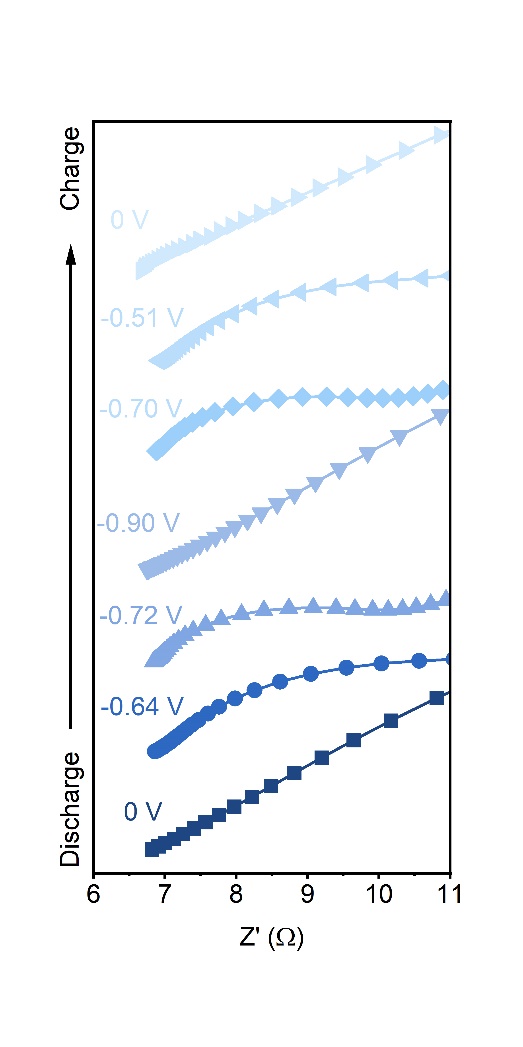


**Fig. S9.** EIS curves in the mid-high frequency regions.

**
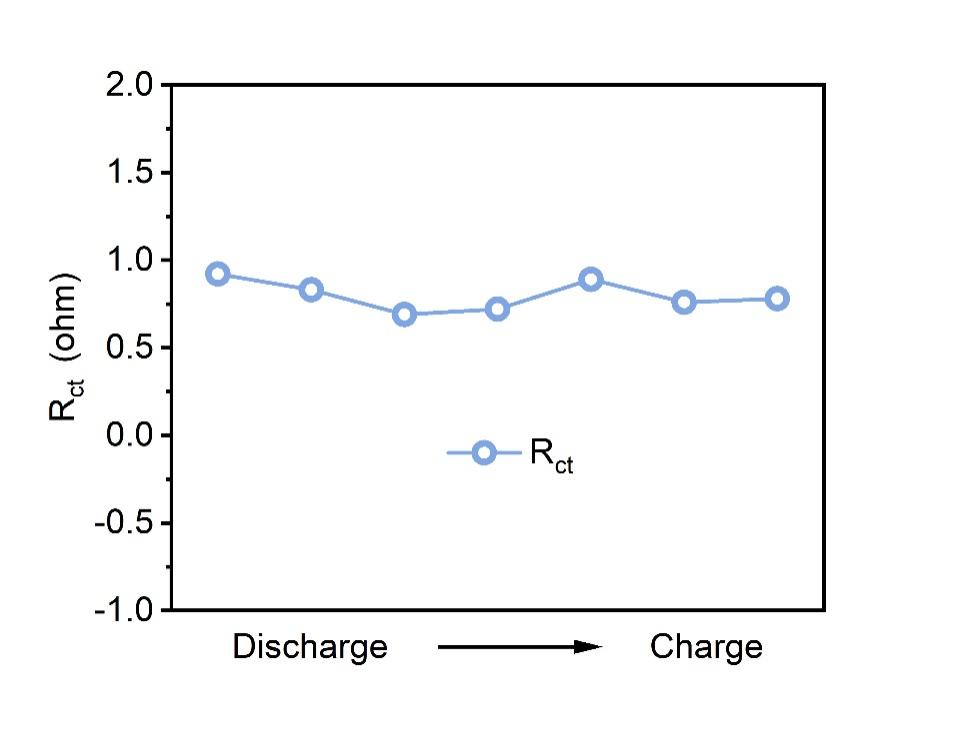
**

**Fig. S10.** R*_ct_* values of NTPI-COF electrode upon Na^+^ uptake/removal.


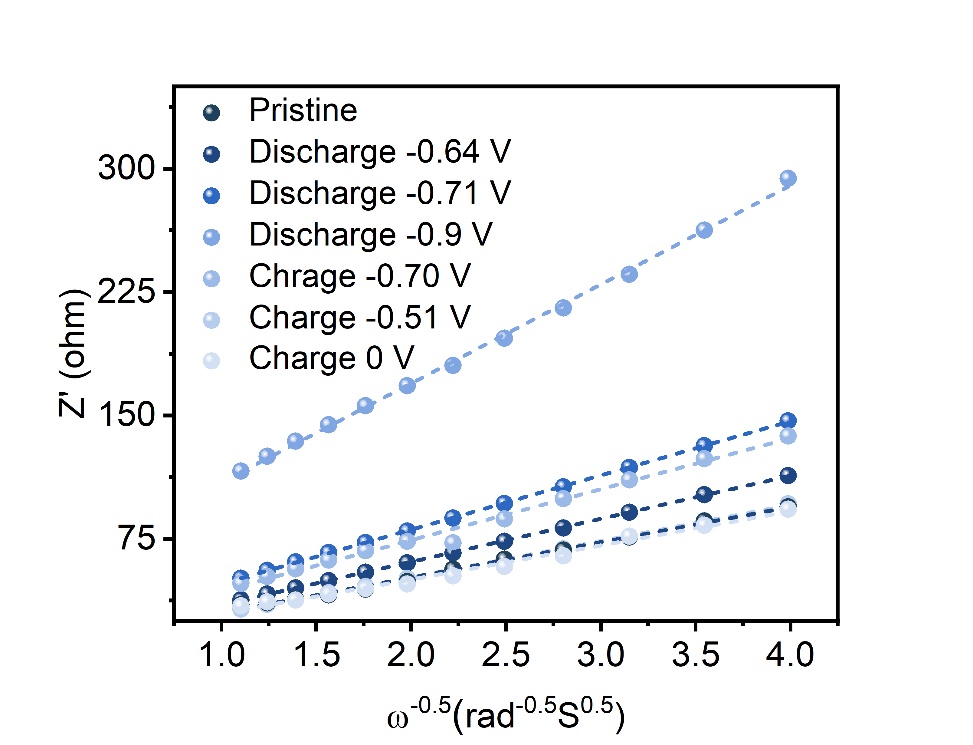


**Fig. S11.** The relationship between Z′ and ω^−0.5^.

The diffusion coefficient (D) can be quantified with the Warburg factor in the low frequency region by deducing from the following equation:

$D=\frac{R^{2}T^{2}}{2\sigma^{2}C^{2}F^{4}n^{4}A^{2}}$ (1)

$Z^{'}=R_{s}+R_{ct}+\sigma\omega^{\frac{1}{2}}$ (2)

Where R is the gas constant, T is the absolute temperature, A is the effective area of electrode, n is the number of transferred electrons per formula unit during discharging, F is the Faraday constant, C is the concentration of Na^+^, respectively. The σ is the Warburg factor, which can be accessed by equation (2).^[5]^


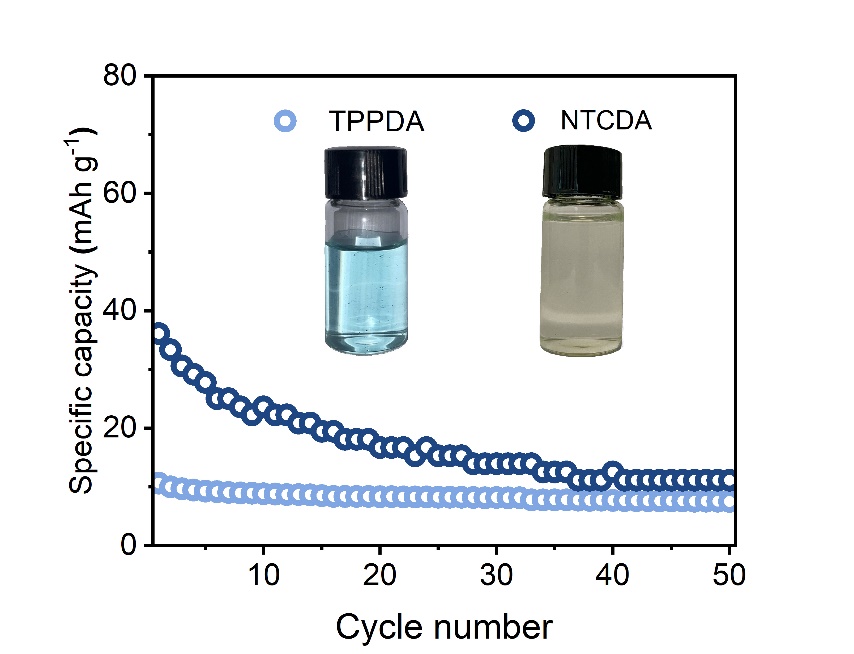


**Fig. S12.** Cyclic properties of NTCDA monomer and TPPDA monomer, state of electrolyte after cycling (inset).


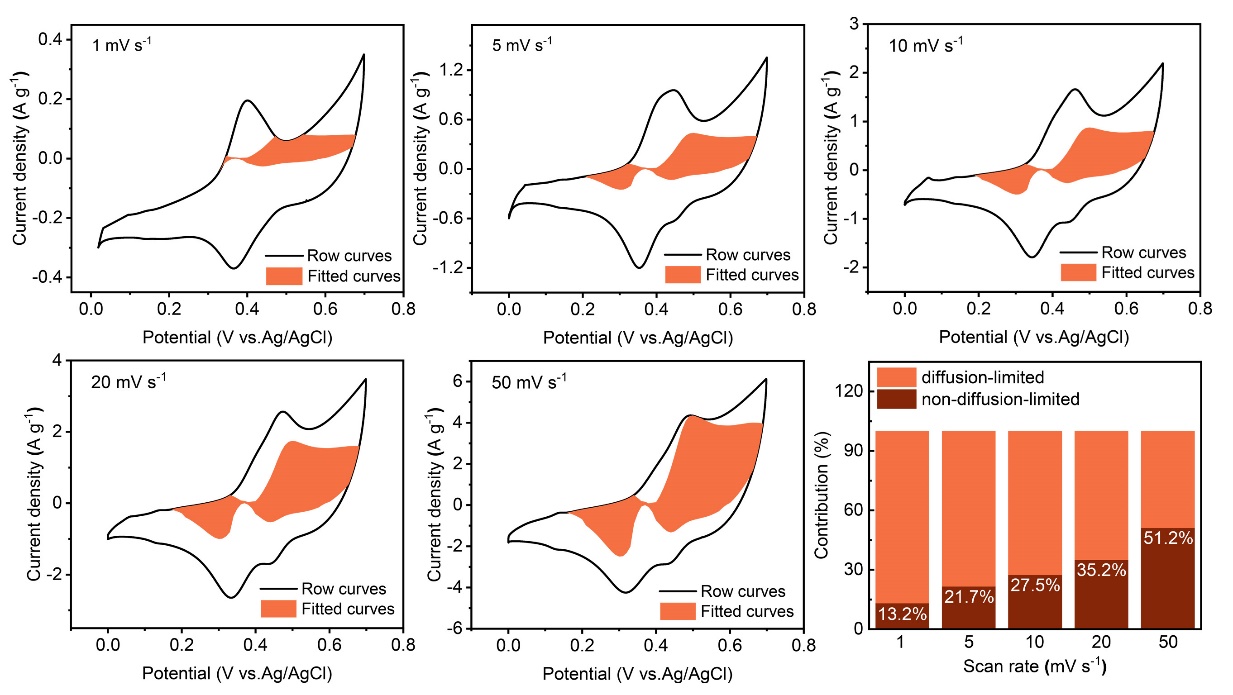


**Fig. S13.** Voltammetric response, diffusion- and capacitive-controlled capacity contribution at different scan rates when NTPI-COF used as cathode.
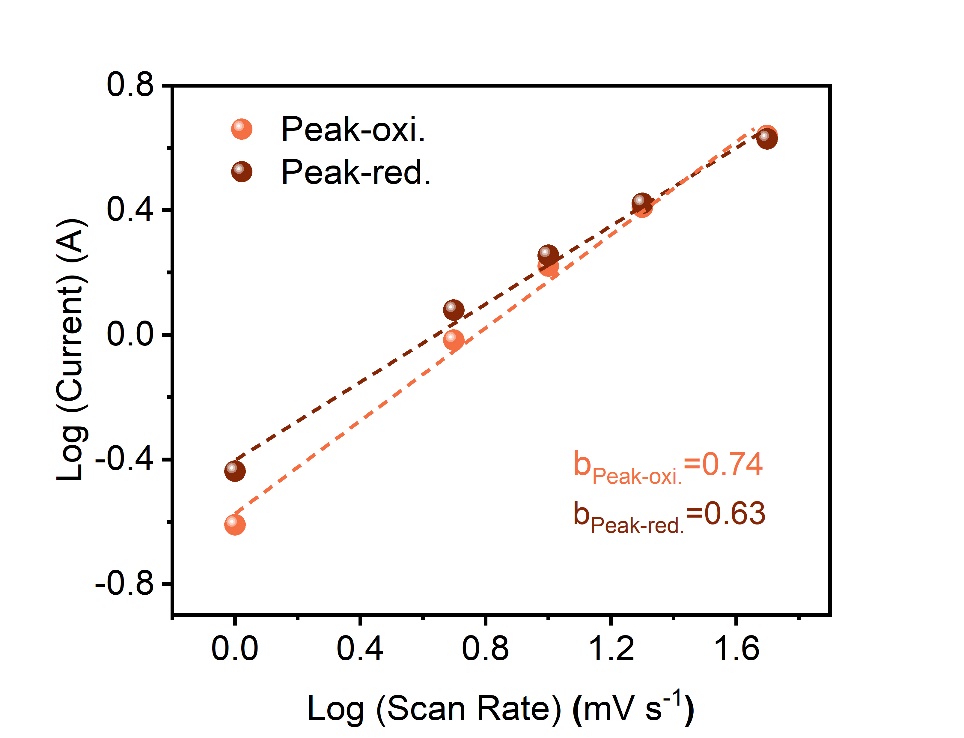


**Fig. S14.** The linear relation of logarithm dependence between peak current densities and scan rates when NTPI-COF used as cathode.


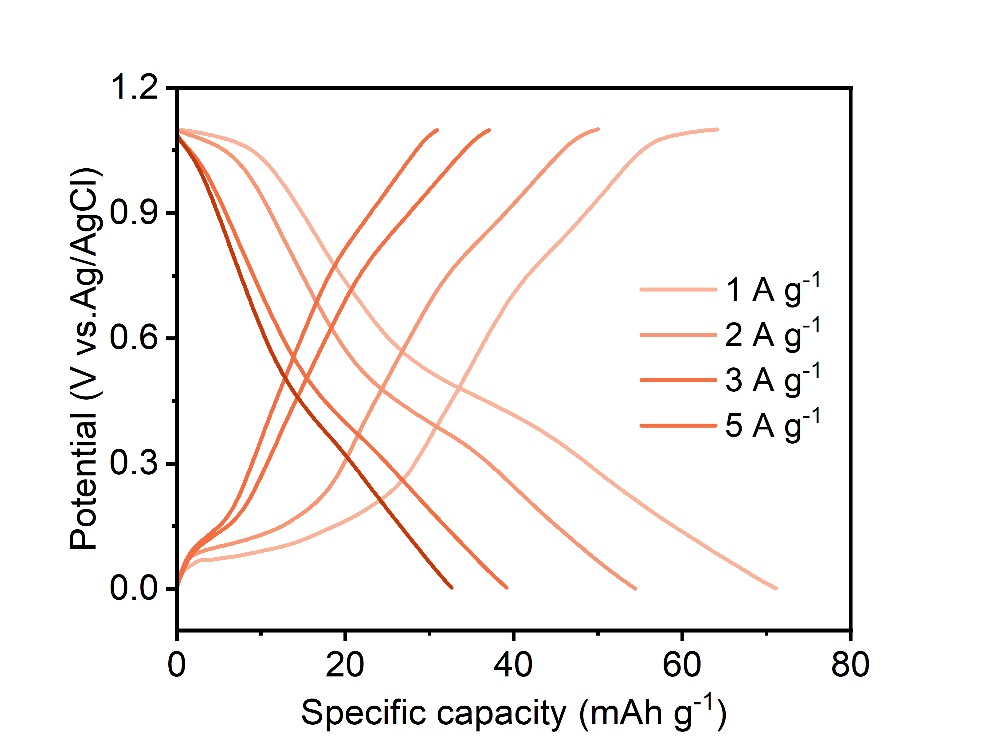


**Fig. S15.** GCD curve of NTPI-COF electrode at positive potential.


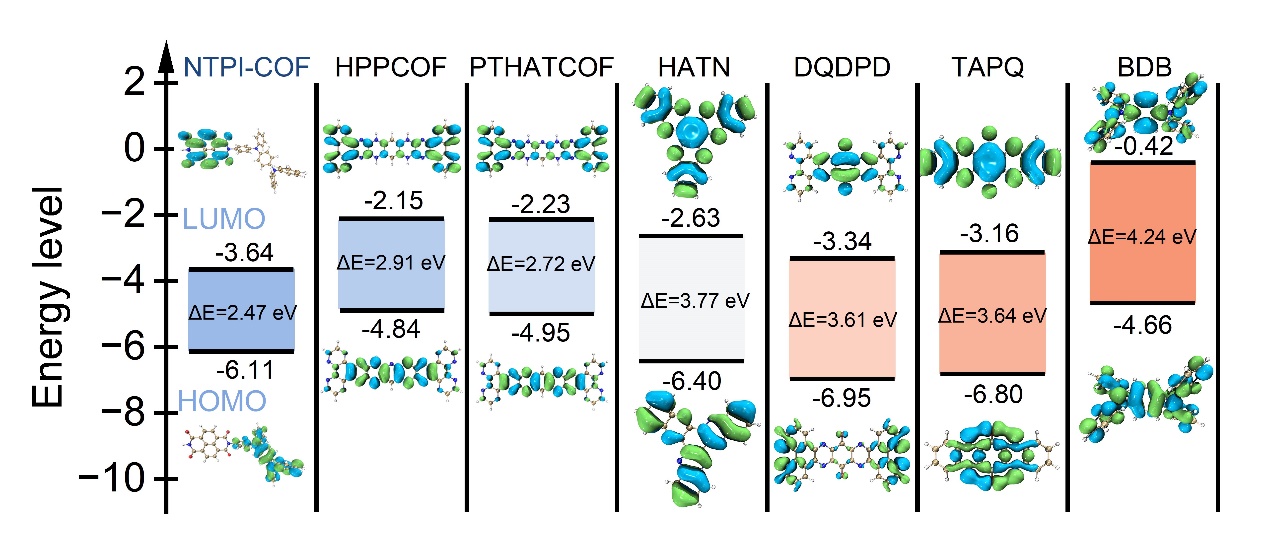


**Fig. S16.** HOMO and LUMO energies of the NTPI-COF compared with other reported organic electrode materials.


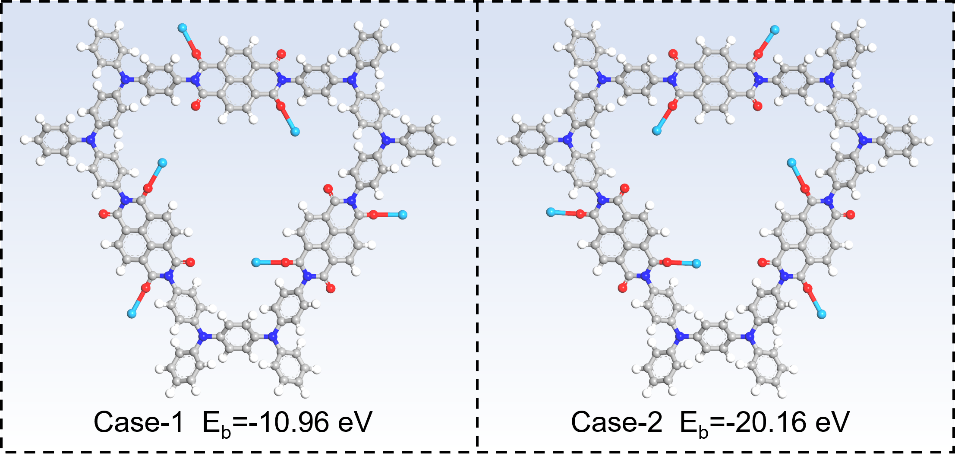


**Fig. S17.** Possible Na-binding geometries and the corresponding binding energies (E_b_) of Na^+^ coordinated with NTPI-COF.

**Supporting References**

1. W. Humphrey, A. Dalke, K. Schulten, *Journal of Molecular Graphics*, **1996**, 14, 33-38.
2. T. Lu, F. Chen, *Journal of Computational Chemistry*, **2012**, 33, 580-592.
3. T. Lu, Q. Chen, *Theoretical Chemistry Accounts*, **2020**, 139, 25.
4. D. Ma, H. Zhao, F. Cao, H. Zhao, J. Li, L. Wang, K. Liu, *Chemical Science*, **2022**, 13, 2385-2390.
5. H. Zhang, L. Yang, P. Zhang, C. Lu, D. Sha, B. Yan, W. He, M. Zhou, W. Zhang, L. Pan, Z. Sun, *Advanced Materials*, **2021**, 33, 2008447.
